# Anomalous Origin of the Right Pulmonary Artery from the Ascending Aorta in an Adult After Prior Patent Ductus Arteriosus Surgery

**DOI:** 10.3390/diagnostics16203318

**Published:** 2026-10-13

**Authors:** Dan Li, Shuo Liang, Hong Zhang

**Affiliations:** 1 Clinical School of Thoracic, Tianjin Medical University, Tianjin 300070, China; 18348908211@163.com; 2 Department of Radiology, Chest Hospital, Tianjin University, Tianjin 300222, China

**Keywords:** anomalous origin of the right pulmonary artery, patent ductus arteriosus, adult congenital heart disease, computed tomography angiography, multiplanar reconstruction, cinematic volume rendering

## Abstract

We present an adult patient with a history of surgical treatment of a patent ductus arteriosus (PDA) in whom contrast-enhanced chest computed tomography (CT) demonstrated anomalous origin of the right pulmonary artery (RPA) from the ascending aorta. Axial CT images clearly demonstrate an discontinuity between the main pulmonary artery and the right pulmonary artery (RPA), and delineate an abnormal opening on the ascending aorta; multiplanar reconstruction technology precisely locates the origin of the artery; dynamic volume imaging integrates the three-dimensional anatomical structure into a model nearly equivalent to a surgical perspective. This case illustrates that an anomalous pulmonary artery associated with a ductus may remain unrecognized at the time of ductus surgery and persist into adulthood, and that a single contrast-enhanced CT examination with multiplanar and cinematic reconstruction provides comprehensive, single-session confirmation of the diagnosis, together with the anatomic roadmap required for surgical planning. For any patient undergoing PDA closure—particularly when pulmonary hypertension or right-heart dilation seems disproportionate—deliberate verification of the origin of both pulmonary arteries may prevent a missed lesion and a second, avoidable operation.

**Figure 1:**
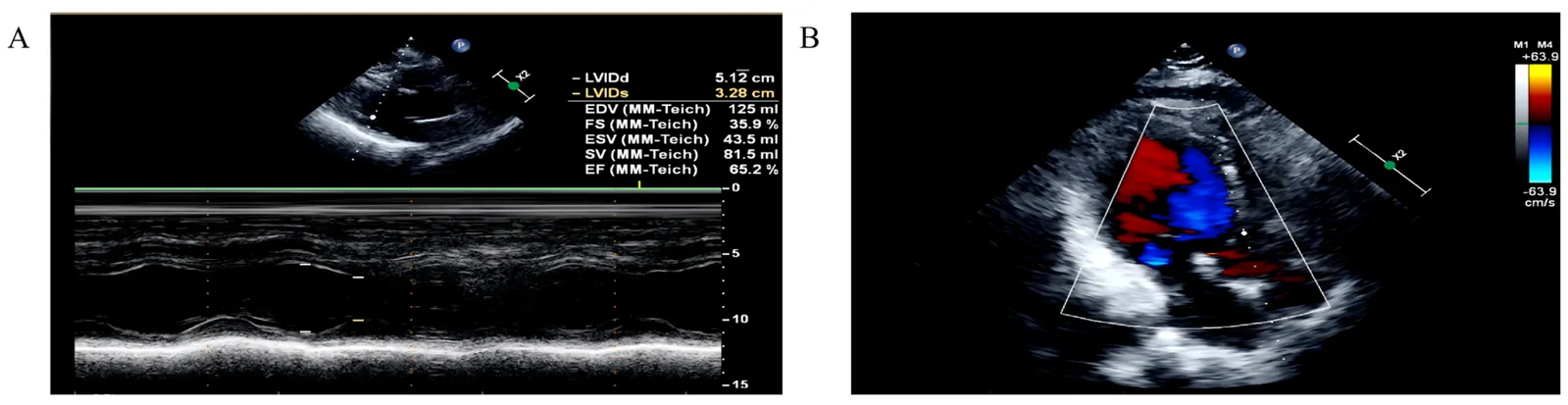
(**A**,**B**) We report a case of a patient who was found to have the right pulmonary artery originating anomalously from the ascending aorta during evaluation for paroxysmal palpitations. The patient had undergone surgical correction for patent ductus arteriosus (PDA) decades prior, and this anomalous vascular origin was not identified during the previous operation. Her clinical course has two unusual characteristics: despite previous surgery being performed in the same anatomic region, the anomalous lesion went undetected; additionally, the patient is now 54 years old, and only has mild pulmonary hypertension. We discuss the potential mechanisms underlying missed diagnosis of this anomalous lesion during PDA surgery, the physiological processes that allowed this patient to survive into adulthood with near-normal pulmonary artery pressure, and the implications of this case for preoperative evaluation of patients undergoing patent ductus arteriosus occlusion. This is a 54-year-old female patient who is well-developed, and presents no thoracic deformity, no cyanosis, and no finger clubbing. The patient has a medical history of hypertension and heart failure. After undergoing surgical treatment for PDA, the patient has maintained regular outpatient follow-ups and received long-term conservative pharmacotherapy, and reports no obvious clinical symptoms. During the follow-up two years ago, an echocardiographic examination found no shunt abnormalities at the arterial level; the anteroposterior diameter of the right ventricle was 14 mm, the basal left-right diameter of the right ventricle was 34 mm, the systolic pulmonary artery pressure was 30 mmHg, the left ventricular end-diastolic volume was 125 mL, the left ventricular end-systolic volume was 43.5 mL, the stroke volume was 81.5 mL, the fractional shortening was 35.9%, and the ejection fraction was 65.2%. Two years ago, echocardiography revealed no significant abnormal shunting at the arterial level; all atrial and ventricular diameters were within normal ranges; the internal diameter of the ascending aorta was normal; the pulmonary artery diameter was widened. LVIDd: left ventricular end-diastolic diameter; LVIDs: left ventricular end-systolic diameter; EDV: end-diastolic volume; ESV: end-systolic volume; SV: stroke volume; FS: left ventricular fractional shortening; EF: left ventricular ejection fraction.

**Figure 2:**
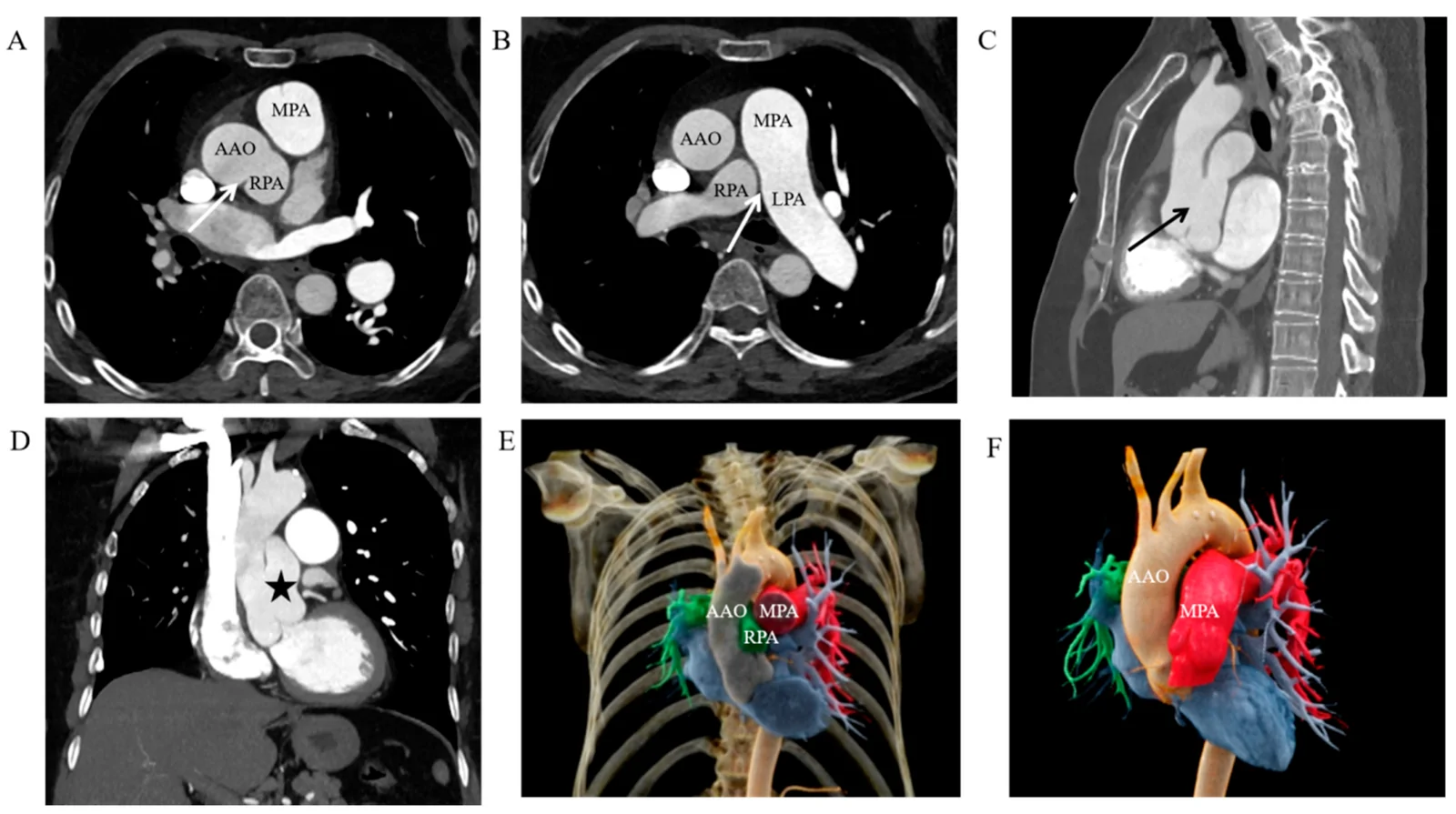
To identify the root cause of the patient’s palpitations, a chest computed tomography (CT) scan was performed. Axial CT images showed that the right pulmonary artery (RPA) originated from the posterior wall of the ascending aorta (AAO) and traveled to the right hilum independently of the main pulmonary artery (MPA), while the MPA continued as the left pulmonary artery (LPA), with no continuity between the MPA and the RPA (**A**,**B**). The MPA was dilated, with an approximate diameter of 36 mm, and the ascending aorta measured approximately 33 mm in diameter. Oblique sagittal multiplanar reconstruction (MPR) clearly revealed the independent opening of the anomalously originated right pulmonary artery on the aortic wall (**C**), while oblique coronal multiplanar reconstruction showed this vessel extending toward the right hilum (**D**). Cinematic volume rendering technique (cVRT) reconstruction confirmed that the right pulmonary artery originated from the aorta, and the main pulmonary artery was continuous with the left pulmonary artery. This reconstruction presented the three-dimensional anatomical structure in a view close to the surgical perspective (**E**,**F**). These imaging findings established the diagnosis of proximal anomalous origin of the right pulmonary artery from the ascending aorta (AORPA). (**A**) Axial chest CT images show that the right pulmonary artery originates from the ascending aorta (indicated by the white arrow). (**B**) The image shows that the right pulmonary artery is independent of the main pulmonary artery (as indicated by the white arrow). (**C**,**D**) Multiplanar reconstruction (MPR) images generated from the same CT dataset demonstrate that the oblique sagittal MPR image reveals an abnormal opening of the right pulmonary artery (RPA, indicated by the black arrow). The oblique coronal MPR image reveals an abnormal RPA (as indicated by the black stars) extending toward the right hilum, confirming a distinct main pulmonary artery (MPA)-left pulmonary artery (LPA) axial relationship. (**E**,**F**) This is a volumetric rendering (cVRT) reconstruction image generated from the same CT dataset. In the sagittal projection view, red marks the main pulmonary artery and left pulmonary artery, green indicates the abnormally originating right pulmonary artery, and blue denotes the pulmonary vein. The image confirms that the ascending aorta (AAO) gives rise to the abnormal right pulmonary artery (RPA), while the main pulmonary artery (MPA) continues as the left pulmonary artery (LPA).

Anomalous origin of the pulmonary artery from the ascending aorta (AOPA) is a rare congenital heart disease with an incidence of approximately 0.12% [1]. This anomaly more commonly affects the right pulmonary artery than the left, and affected patients frequently have additional cardiovascular malformations, among which patent ductus arteriosus (PDA) is the most prevalent in the right-sided type. Most patients with AOPA develop symptoms shortly after birth, and the natural course of the disease lasts no more than 1 year in over 80% of cases [2]. Therefore, diagnosis in adulthood is extremely rare; the few reported adult cases illustrate a distinctly different clinical presentation at a later age. This case report presents an adult case of anomalous origin of the right pulmonary artery from the ascending aorta (AORPA), distinguished by the patient having no significant clinical symptoms for a prolonged period after PDA surgery and never developing severe pulmonary hypertension (PH).Several reasonable mechanisms can explain why no abnormal vascular origin was detected in the patient during PDA surgery and long-term follow-up. First, several decades earlier, CT was not routinely performed before arterial duct ligation. There was almost no opportunity to detect this abnormality during surgical exposure. Classic ligation of patent ductus arteriosus via left posterolateral thoracotomy exposes the aortic isthmus and the arterial duct, but does not reveal the intrapericardial relationship between the ascending aorta and the main pulmonary artery. Unless an abnormal origin is suspected preoperatively, there is no justification to explore this anatomical site. Relevant precedents have been documented: Yang et al. [3] described a 22-year-old female patient in whom the aortic origin of the right pulmonary artery was missed when she underwent patent ductus arteriosus ligation 10 years earlier, requiring reoperation in adulthood, a process highly similar to the situation of our patient. Second, the diagnostic anchoring effect may also play a role: once the presence of a patent ductus arteriosus is confirmed, it becomes easy to attribute all clinical manifestations to this finding, and postoperative symptom relief after ligation can further mask the presence of residual lesions. Yang et al. [3] also pointed out that aortic origin of the right pulmonary artery is easily misdiagnosed as patent ductus arteriosus. The case of Cao et al. illustrates the cost of delayed identification: their patient with aortic origin of the right pulmonary artery combined with patent ductus arteriosus progressed to inoperable severe pulmonary hypertension, presenting with right-to-left shunt and cyanosis [4]. Third, during long-term follow-up, the patient underwent echocardiography, and transthoracic echocardiography has a well-recognized blind spot for the origin of pulmonary artery branches: the posterior origin location close to the aortic valve is within the limited acoustic window. In a multi-detector spiral CT study including 19 patients, the rate of misdiagnosis or missed diagnosis of anomalous aortic origin of the pulmonary artery by echocardiography reached 21% [5], and anomalous aortic origin of the pulmonary artery was once misdiagnosed as isolated patent ductus arteriosus [6].It has been suggested that reactive vasoconstriction and vascular remodeling in the lung perfused by the aorta can limit shunt flow and provide partial protection to the pulmonary circulation, which may partially explain why a small proportion of patients survive to adulthood with only mild pulmonary hypertension. The differing Heath–Edwards grades between the two lungs supports the conclusion that vascular disease does not progress uniformly [7]. A 43-year-old unoperated survivor has been described [8] as has an adult with left hemitruncus and normal right-sided pressures [9]; our patient appears to sit at the favorable end of this spectrum. Her long-standing hypertension and the age-related decline in left ventricular compliance might have disturbed a previously stable equilibrium and could plausibly account for the onset of palpitations, although this remains speculative. To date, most reported adult cases of anomalous origin of the pulmonary artery from the aorta present with hemoptysis, dyspnea, or heart failure, and suffer from moderate-to-severe pulmonary hypertension [10,11,12,13,14]; the recorded pressures are sometimes extremely high, with the mean pressure of the right pulmonary artery reaching 92 mmHg, the right ventricular systolic pressure of patients with Eisenmenger physiology being 150 mmHg, and the pulmonary artery systolic pressure being 111 mmHg. Several patients received conservative treatment because the pulmonary vascular disease made repair impossible, or the patients refused surgery. A small number of patients have more favorable hemodynamic characteristics: elevated pressure is only limited to the anomalous artery, with no contralateral pulmonary hypertension, which can be relieved by ligation [15]; origin stenosis plays a protective role in the anomalous lung, while severe pulmonary hypertension exists in the normally connected lung [16]; long-term survival is accompanied by pulmonary arteriovenous fistula [17]. To the best of our knowledge, there are few reported adult patients who have no obvious symptoms and do not develop severe pulmonary hypertension for decades after the lesion is missed; this case was discovered incidentally, with an estimated pulmonary artery systolic pressure of 30 mmHg, which distinguishes it from most cases documented in the literature in this field. Surgical intervention may be considered in the early stages of the disease before pulmonary hypertension develops. The surgical approach involves dissecting the main pulmonary artery with an ectopic origin and reconstructing it to restore normal anatomical relationships. The procedure typically involves transecting the abnormally originating vessel at its opening and performing direct anastomosis with the pulmonary artery. For patients who cannot undergo surgical correction, thoracotomy for combined cardiopulmonary transplantation may be considered [18]. For the patients in this study, the repair procedure is technically feasible, and this treatment option has been presented to the patients. As the family members refused the surgery, a joint decision was made to postpone the operation after multidisciplinary consultation. The basis for this decision is that the patient’s pulmonary hypertension is mild, the functional status remains good, the lesion has been tolerated for more than 50 years, and the surgical risk for a 54-year-old patient cannot be ignored. The prognosis of adult patients who do not undergo surgery is still unclear, but not necessarily very poor: there are relevant records of long-term survival [8], and there are also reports showing that the condition improved after taking tadalafil under monitoring [10]. Meanwhile, there are also relevant reports of adult patients who did not undergo surgery developing advanced progression of pulmonary hypertension, hemoptysis, pulmonary artery aneurysm, and pulmonary arteriovenous fistula; so, continuous monitoring seems to be a prudent practice. This case also reveals a novel and instructive dimension: when clinical attention is focused on the coexisting patent ductus arteriosus, an abnormal right pulmonary artery (RPA) may be missed. A recent report described two adult patients who had previously undergone isolated PDA ligation ten years before presentation. In these cases, the accompanying abnormal right pulmonary artery (RPA) was overlooked, ultimately requiring reoperation in adulthood—a scenario highly similar to our case. This experience demonstrates that for all patients referred for PDA closure surgery, particularly those presenting with pulmonary hypertension or asymmetric right heart dilation, the origin sites of both pulmonary arteries must be unequivocally confirmed. The use of single-slice enhanced CT examination, which integrates axial images, multiplanar reconstruction (MPR), and volume rendering technology (cVRT) data, enables comprehensive, three-dimensional visualization of any remaining abnormal anatomical structures of the right pulmonary artery after surgery. This method is an indispensable non-invasive imaging tool for clinical diagnosis, preoperative planning, and long-term postoperative follow-up.

## Data Availability

No new data were created or analyzed in this study. Data sharing is not applicable to this article.

## Abbreviations

AAO	Ascending aorta
AOPA	Anomalous origin of one pulmonary artery from the aorta
AORPA	Anomalous origin of the right pulmonary artery from the ascending aorta
CT	Computed tomography
cVRT	Cinematic volume rendering
LPA	Left pulmonary artery
MPA	Main pulmonary artery
MPR	Multiplanar reconstruction
RPA	Right pulmonary artery
EDV	End-diastolic volume
EF	Left ventricular ejection fraction
ESV	End-systolic volume
SV	Stroke volume
FS	Left ventricular fractional shortening
PDA	Patent ductus arteriosus
PH	Pulmonary hypertension
LVIDd	Left ventricular end-diastolic diameter
LVIDs	Left ventricular end-systolic diameter
